# Acute Diverticulitis in the Young: The Same Disease in a Different Patient

**DOI:** 10.1155/2013/867961

**Published:** 2013-03-10

**Authors:** Adolfo Pisanu, Valentina Vacca, Isabella Reccia, Mauro Podda, Alessandro Uccheddu

**Affiliations:** Clinica Chirurgica, Department of Surgery, University of Cagliari, Azienda Ospedaliero-Universitaria, Presidio Policlinico di Monserrato, Blocco G, SS 554 km 4,500, 09042 Monserrato (CA), Italy

## Abstract

*Background*. Natural history and risk factors for diverticulitis in young patients are still debatable. This study aimed to assess whether difference exists in patients aged 50 and younger when compared to older patients and to identify risk factors for acute diverticulitis in the young. *Patients and Methods*. From January 2006 to December 2011, 80 patients were admitted to our department for acute diverticulitis. We carried out a cross-sectional study in 23 patients (28.7%) aged 50 and younger and 57 older patients (71.3%). *Results*. Acute diverticulitis in the young was not more aggressive than in the older patient. Diverticulitis at patient's admission was similar with respect to Hinchey's stage and prior history of diverticulitis. No significant difference was found for both medical and surgical treatment. The rate of recurrent diverticulitis in nonoperated patients was similar. Male gender, body mass index ≥25, and assumption of alcohol were independent risk factors for the occurrence of an acute diverticulitis in the young. *Conclusions*. The same disease seems to be affecting young patients such as overweight or obese male individual. Current policies to prevent diverticular disease and its related complications must include obesity control together with high-fiber diet and regular exercise.

## 1. Introduction

The development of colonic diverticula is common in Western industrialized countries and it is usually associated with a low-fiber diet [1]. Diverticular disease affects more than 65% of people over the age of 80, whereas less than 10% of people younger than 40 will develop the disease [1, 2]. Acute diverticulitis occurs in 10%–25% of patients with diverticular disease, as it is the most frequent complication [3, 4]. Recent studies have reported an increasing incidence of acute diverticulitis in hospitalized individuals younger than the age of 50, ranging from 18% to 34% [5–7]. This new trend and changing pattern of the disease in young patients have been clearly demonstrated by recent epidemiological studies on the variation in hospital admission for diverticulitis and geographic distribution [6, 8, 9]. However, the natural history and the risk factors for diverticulitis in young patients are still a matter to be debated. Although some authors reported more severe clinical courses in young patients with diverticulitis [10–14], others suggested that diverticulitis is not more aggressive in the young patient when compared to the older one [5, 7, 15, 16]. After these uncertainties, there is no consensus about the management of acute diverticulitis in the young with some different options reported in the literature [5, 13, 17, 18].

Currently, there is evidence that obesity and lifestyle factors, other than dietary fiber deficiency, may also be predisposing to colonic diverticular disease [10, 19–21]. Obesity has been considered as risk factor for diverticulitis and diverticular complication such as bleeding, perforation, and recurrent diverticulitis in young male patients [10, 22, 23].

We carried out a cross-sectional study to identify those risk factors for acute diverticulitis and for complicated diverticulitis in the young patient. This study aimed also to assess whether difference exists in patients aged 50 and younger when compared to older patients. The present research was based on a single-institution experience in a relatively short span of time with homogeneity in the clinical management of acute diverticulitis.

## 2. Patients and Methods

Over the period from January 2006 to December 2011, 80 consecutive patients were admitted to our surgical department for acute colonic diverticulitis. These patients represented the case cohort selected for the current study. We carried out a cross-sectional study in 23 patients (28.7%) aged 50 and younger and 57 older patients (71.3%). All the medical records were retrospectively reviewed and the comparison between groups involved evaluation of demographic data (age and sex); body mass index (BMI); health conditions in accordance with the American Society of Anesthesiology (ASA) grading and comorbidities; concomitant use of medication such as aspirin, nonsteroidal anti-inflammatory drugs (NSAIDs) or steroids; smoking habit and alcohol consumption; symptoms at presentation; duration of symptoms; severity of diverticulitis according to Hinchey's classification [24]; prior history of diverticulitis; laboratory signs of inflammation; and medical or surgical treatment. Clinical symptoms and signs made the diagnosis of acute diverticulitis, which was confirmed by ultrasonography and CT scan of the abdomen. Patients with inflammatory or ischemic bowel disease were excluded from the study. Colonoscopy was performed in all nonoperated patients with the diagnosis of acute diverticulitis one month after to exclude other diagnoses such as colonic cancer. Data regarding the followup were collected during the course of an interview by a member of the medical team or by phone. The average duration of the followup in the younger group was 39 months (range 6–77 months, median 43 months) while it was 40 months (range 6–67 months, median 41 months) in the older one. Informed consent was obtained from each patient included in the study, which was approved by our advisory board.

All statistical analyses were carried out using the MedCalc 2011 statistical software (version 11.5.1). Data for age, BMI, duration of symptoms, white blood cell (WBC) count, and hospital stay were presented as median and 95% confidence interval (CI). Data were compared for statistical analysis using the Fisher exact test for small sample to evaluate differences between qualitative variables and using the Student's *t*-test to compare quantitative variables. The aim of the statistical analysis was mainly to identify those independent risk factors for acute diverticulitis and for complicated diverticulitis in the young patient by means of stepwise logistic regression analysis [25]. *P* values have been checked and reported as calculated by the statistical software. Differences were considered significant when *P* < 0.05.

## 3. Results

Our series includes 80 Caucasian patients with acute colonic diverticulitis: 35 females and 45 males with an average age of 60 years (range, 34–87 years). The median of age was significantly different in the two study groups: 43 years in the younger group versus 67 years in the older one (*P* = 0.001). Male gender was significantly more frequent in patients aged 50 and younger as 74% of them were male versus 49.1% of the older group (*P* = 0.046). No significant difference was found regarding the median of BMI, but younger patients had a BMI higher than 25 (i.e., overweight) in 91.3% of cases versus 63.1% of older patients and this difference was statistically significant (*P* = 0.024), (Table 1).

A significant difference was found regarding ASA score, which was lower in younger patients (*P* = 0.000), as older patients had significantly more chronic disease, including ischemic heart disease (*P* = 0.001) and hypertension. Patients in both groups were similar in respect to steroids intake and smoking habit. One surgically treated patient of the younger group with a perforated diverticulitis was taking aspirin before hospitalization; eight patients of the older group with acute diverticulitis were taking aspirin or NSAIDs: 3 patients recovered after medical treatment and suspension of anti-inflammatory drugs, one patient had bleeding that resolved spontaneously, another patient had bleeding that required surgical treatment, one other patient was operated on for perforated diverticulitis, and two others were later submitted to surgical treatment. The chronic consumption of alcohol in terms of moderate and occasionally excessive doses was significantly associated with younger patients (*P* = 0.046), (Table 2).

Abdominal pain was always felt in the left iliac fossa or diffuse in the abdomen in the older group, while it was felt in the right iliac fossa and hypogastrium in 21.7% of younger patients (*P* = 0.001). There were no differences between groups about other symptoms at presentation. The duration of symptoms was significantly shorter in the younger group than in the older one (median of 24 versus 48 hours, resp., *P* = 0.009). The disease was similar in both groups with respect to Hinchey's stage and prior history of diverticulitis. Clinical data were insufficient to show how many patients had a prior history of irritable bowel disease.

The median value of WBC count was significantly higher in the younger group than in the older one (12700 versus 12000/mm^3^, *P* = 0.002); leukocytosis on admission (≥ 10500/mm^3^) was significantly associated with diverticulitis in younger patients (*P* = 0.020), (Table 3). No significant differences were found for both medical and surgical treatments. Eight younger patients (34.8%) required surgical intervention versus 19 older patients (33.3%) without significance difference (Table 4). Medically treated diverticulitis resolved in all cases by keeping patients on fluids and antibiotics for at least seven days.

No significant differences were found with respect to the median of hospital stay between groups. Two postoperative deaths related to septic shock and multiple organ failure syndrome from fecal peritonitis occurred in the older group (Table 5). No different diagnosis was made after colonoscopy one month after the acute event and no colonic malignancy was detected in both groups (data not shown). On the followup, the rate of recurrent diverticulitis in nonoperated patients was similar in both groups. Two patients of the older group died for unrelated causes. Four patients of the older group were lost at followup (Table 5).

Multivariate analysis showed that male gender, BMI ≥ 25, and assumption of alcohol were independent risk factors for the occurrence of an acute diverticulitis in the young (odds ratio 2.93, 6.12, and 3.14, resp.) (Table 6(a)). However, logistic regression failed to demonstrate that the younger age is a risk factor for complicated diverticulitis. The only factors retained in the multivariate statistical model as predictive for complicated diverticulitis (Hinchey III and IV) were fever (defined as body temperature >38°C) and leukocytosis (>12000/mm^3^), regardless of age (odds ratio 3.05 and 3.55, resp.) (Table 6(b)).

## 4. Discussion

Current scientific evidence shows that decreased fiber intake and chronic changes in colonic motility are involved in the development of diverticula [4, 6]. More recently, obesity has also been associated to higher incidence of diverticulitis and its related complications in young male individuals [7, 10, 19, 21, 26, 27]. Our investigation clearly demonstrated that the occurrence of an acute diverticulitis was significantly associated with young male patients with a BMI ≥ 25 and chronic consumption of alcohol in terms of moderate and occasionally excessive doses. However, our study failed to demonstrate a correlation between BMI and the incidence of complicated diverticulitis as reported by other authors [22, 23]. The prevalence of obesity in these young patients was as high as 91.3%, which was higher than the current trend of overweight and obesity in Italy [28, 29]. More than 50% of Italian adult men and about 1 of 3 women are overweight or obese [29]. Interestingly, although the average BMI of Sardinian people is similar to that of other Italian regions, there is an increasing prevalence of overweight and obesity among male Sardinian children and adolescents [30].

To the best of our knowledge, this is the first study that showed a correlation between chronic consumption of alcohol and the occurrence of an acute diverticulitis. Other authors reported that alcohol intake was not associated with increased risk of symptomatic diverticular disease [31]. Further studies are needed to clarify this epidemiological aspect.

In the current study, univariate analysis showed some clinical differences between the two groups of age. Abdominal pain was always felt in the left iliac fossa or diffuse in the abdomen in the older group, while it was felt in the right iliac fossa and hypogastrium in 21.7% of younger patients. This is possibly due to the variable localization of the sigmoid colon in young patients as reported by other authors [21], but this clinical finding was not related to younger age at multivariate analysis.

The duration of symptoms of diverticulitis was significantly shorter in the younger group than in the older one. This result may be related to the lower sensitivity to the symptoms of the disease, which are frequently encountered in elderly people with diffuse peritonitis as being responsible for the delay in diagnosis and hospitalization [32].

In the literature, there is a significantly increased frequency of diverticular disease in patients with irritable bowel syndrome [33]. Our investigation failed to demonstrate this relationship both in the young and in the elderly patient due to the insufficient clinical data.

Regular use of aspirin or NSAIDs has been strongly related to an increased risk of diverticulitis and diverticular bleeding [34]. However, these findings have clinical implications especially in elderly patients as seen in our experience.

Mean WBC count was significantly higher in the younger group than in the older one. Leukocytosis on admission (≥10500/mm^3^) was significantly associated with diverticulitis in the young. These results may indicate a more intense inflammatory and cytokine response in younger patients because of the higher prevalence of overweight and obesity, the adipose tissue being responsible for an increased secretion of cytokines and alterations of the intestinal microflora [19]. However, the only factors retained in the multivariate statistical model as predictive for complicated diverticulitis were fever and leukocytosis, regardless of age.

Overall, in our series acute diverticulitis in the young patient was not more aggressive than in the older one. Age itself was not a risk factor for the occurrence of a complicated diverticulitis. Diverticular disease at patient's admission was similar in both groups of age with respect to Hinchey's stage and prior history of diverticulitis. However, no significant difference was found for both medical and surgical treatment and the rate of recurrent diverticulitis in nonoperated patients was similar in both groups.

Our results are consistent with the findings of other studies that have found no evidence for more virulent disease in younger patients [7, 10, 16, 21, 35–37]. In the American Society of Colon and Rectal Surgeons practice guidelines, there is no consensus as to whether young patients have an increased risk of complicated or recurrent diverticulitis [18]. Because life span is potentially longer in young patients, the risk of recurrence disease may be higher than in the older population [18, 21]. Therefore, any increased risk is a chronologic rather than pathologic phenomenon [38]. Acute diverticulitis in the young could be managed as in the older patients [16], the indication for surgery being not dependent on age but on the actual clinical findings [39].

## 5. Conclusions

In our series, diverticular disease and its related complications were not a different disease in both groups of age. However, young patients with acute diverticulitis seemed to be different patients. In recent years, we have observed a changing pattern of diverticular disease because of an increasing rate of hospital admission of younger patients [8]. Epidemiological investigations clearly demonstrated that this new trend is correlated with overweight and obesity in children and young adults [6, 26]. Thus, the same disease seems to affect different patients such as overweight or obese young male individuals. Current policies to prevent diverticular disease and its related complications must include overweight and obesity control together with high-fiber diet and regular exercise.

## Figures and Tables

**Table 1:** Patients' characteristics.

Patients	≤50 years	>50 years	*P*
Number	23	57	
Sex (male/female)	3.5/1.0	1.0/1.1	0.046
Age (years), median (95% CI)	43 (39.01–45.00)	67 (64.00–70.21)	0.001
Range (years)	33–50	51–87	
BMI, median (95% CI)	26.2 (25.88–28.23)	26 (24.31–27.06)	0.302
Range	21–46	19.5–37.5	
BMI ≥ 25	21/23 (91.3%)	36/57 (63.1%)	0.024

CI: confidence interval; BMI: body mass index.

**Table 2:** Patients' comorbidities and habits.

Parameter	≤50 years	>50 years	*P*
Number	23	57	
ASA			
1-2	23 (100.0%)	37 (65.0%)	0.000
3-4	0	20 (35.0%)	
Hypertension	2 (8.6%)	27 (47.3%)	0.001
COPD	0	4 (7.0%)	0.319
Ischemic heart disease	1 (4.3%)	12 (21.0%)	0.095
Diabetes mellitus	0	6 (10.5%)	0.175
Aspirin or NSAIDs	1 (4.3%)	8 (14%)	0.434
Steroids	1 (4.3%)	2 (3.5%)	0.637
Smoke	13 (56.5%)	20 (35.0%)	0.087
Alcohol	17 (73.9%)	27 (47.3%)	0.046

ASA: American Society of Anesthesiology; COPD: chronic obstructive pulmonary disease.

NSAIDs: nonsteroidal anti-inflammatory drugs.

**Table 3:** Patients' symptoms, signs, and complications.

Parameter	≤50 years	>50 years	*P*
Number	23	57	
Site of pain			
Lower left quadrant and hypogastrium	18 (78.3%)	35 (61.4%)	0.195
Lower left quadrant and diffuse	—	22 (38.6%)	0.000
Lower right quadrant and hypogastrium	5 (21.7%)	—	0.001
Stypsis	3 (13%)	10 (17.5%)	0.747
Diarrhea	0	6 (10.5%)	0.175
Dysuria	0	2 (3.5%)	0.906
Tenesmus	0	2 (3.5%)	0.906
Fever > 38°C	13 (56.5%)	19 (33.3%)	0.078
Bleeding	1 (4.3%)	5 (8.7%)	0.833
Severe diverticulitis	15 (65.2%)	35 (61.4%)	0.804
Hinchey 1-2	2 (8.6%)	7 (12.3%)	0.945
Hinchey 3-4	6 (26.0%)	15 (26.3%)	0.795
Duration of symptoms (hours), median (95% CI)	24 (23.78–48.00)	48 (31.80–49.15)	0.009
Range (hours)	20–96	10–120	
Duration > 24 hours	12 (52.1%)	27 (47.3%)	0.279
Prior history of diverticulitis	(30.4%)	28 (49.1%)	0.332
First episode	16 (69.6%)	29 (50.9%)	
WBC/mm^3^, median (95% CI)	12700 (11960.33–14519.00)	12000 (9956.47–14259.17)	0.002
Range	5500–17300	2100–28400	
WBC > 10500/mm^3^	20 (86.9%)	34 (59.6%)	0.020

CI: confidence interval; WBC: white blood cell.

**Table 4:** Surgical and medical treatment.

Patients	≤50 years	>50 years	*P*
Number	23	57	
Medical treatment	15 (65.2%)	38 (66.7%)	0.891
Surgery	8 (34.8%)	19 (33.3%)	
Anterior resection	3 (13.0%)	6 (10.5%)	0.257
Hartmann operation	5 (21.7%)	9 (15.8%)	0.107
Bridge colostomy	—	4 (7.0%)	0.319

**Table 5:** Hospital stay, postoperative, and followup results.

Parameter	≤50 years	>50 years	*P*
Number	23	57	
Overall mean hospital stay (days) median (95% CI)	10 (8.00–10.01)	10 (9.02–11.00)	0.192
Range (days)	6–14	7–32	
Mean postoperative hospital stay (days) median (95% CI)	9.5 (9.00–13.22)	12 (12.00–14.52)	0.141
Range (days)	9–15	12–30	
Surgical treatment	8/23 (34.8%)	19/38 (33.3%)	0.891
In-hospital mortality	—	2/19 (10.5%)	0.882
Recurrent abdominal pain	1/8 (12.5%)	1/17 (5.5%)	0.825
Medical treatment	15/23 (65.2%)	38/57 (66.7%)	0.891
Recurrent diverticulitis with readmission	3/15 (20.0%)	2/34 (5.9%)	0.160
Recurrent diverticulitis requiring surgery	1/15 (6.6%)	2/34 (5.9%)	0.589
Death for unrelated causes at followup	—	2/55 (3.6%)	NA
Lost at followup	—	4/55 (7.2%)	NA

CI: confidence interval; NA: not applicable.

**Table:** (a)

Parameter	*β* Coefficient	Standard error	*P*	OR	95% CI
Male gender	1.07	0.543	0.047	2.93	1.01–8.51
BMI ≥ 25	1.81	0.789	0.021	6.12	1.30–28.77
Alcohol	1.14	0.543	0.035	3.14	1.08–9.14

**Table:** (b)

Parameter	*β* Coefficient	Standard error	*P*	OR	95% CI
Fever > 38°C	1.11	0.480	0.020	3.05	1.18–7.82
WBC > 12000	1.26	0.516	0.013	3.55	1.29–9.79

OR: odds ratio; CI: confidence interval; WBC: white blood cell; and BMI: body mass index.
